# Angiogenesis after acute myocardial infarction: a bibliometric -based literature review

**DOI:** 10.3389/fcvm.2025.1426583

**Published:** 2025-02-13

**Authors:** Yu Tan, Min Li, Xiaojuan Ma, Dazhuo Shi, Wei Liu

**Affiliations:** ^1^ Department of Cardiology, Beijing Jishuitan Hospital, Capital Medical University, Beijing, China; ^2^ National Clinical Research Center for Chinese Medicine Cardiology, Xiyuan Hospital, China Academy of Chinese Medical Sciences, Beijing, China

**Keywords:** angiogenesis, acute myocardial infarction, bibliometric, VOSviewer, CAD

## Abstract

**Objective:**

The prevalence of acute myocardial infarction, a severe ischemic cardiac disease, is on the rise annually. The establishment of coronary collateral circulation in the border zone of the infarct can effectively relieve myocardial ischemia and impede cell death, while angiogenesis can promote the formation of collateral circulation in the ischemic tissues. Over the past two decades, studies related to angiogenesis in acute myocardial infarction have increased rapidly. However, there is a lack of bibliometric studies in this particular field.

**Methods:**

For this study, we employed bibliometric analysis to outline focal points and patterns in scientific and clinical research. The collection of literature was gathered using the Web of Science Core Collection database. Bibliometric and visual analysis were conducted. Knowledge maps were generated using CiteSpace and VOSviewer software.

**Results and conclusions:**

With the deepening of the research, therapeutic angiogenesis will become a treatment direction for acute myocardial infarction in the future.

## Introduction

Acute myocardial infarction (AMI) is a serious cardiovascular disease caused by rupture or erosion of an unstable atherosclerotic plaque, which trigger fatally thrombus obstruction of the lumen of the offender's coronary artery, ultimately leading to progressive myocardial tissue necrosis in the hypoperfused blood supply region (1). AMI remains one of the leading causes of cardiovascular death worldwide, as well as a major cause of chronic heart failure, severely affecting patients' life expectancy and long-term quality of survival (2). Currently, the application of secondary prevention agents for coronary artery disease and the rapid development of percutaneous coronary intervention (PCI) techniques have improved the survival of AMI patients and reduced the rate of recurrent MI (3). However, despite the prevalence of accessible and stringent in-time reperfusion therapy and advanced antithrombotic strategies, patients with severe left cardiac insufficiency remains intractable to be managed (4). These individuals have a very high probability of heart failure, which severely impairs the long-term prognosis (5). Therefore, additional therapeutic strategies for post-AMI are urgently needed.

In recent years, the mechanisms of angiogenesis in infarct border zone have attracted interest in the cardiovascular field. And researchers aim to explore new therapeutic directions for improving impaired cardiac function after myocardial infarction. Lumen occlusion of epicardial coronary arteries triggers irreversible damage to the coronary microcirculation, ultimately resulting in vascular disintegration within the infarct core (6). Myocardial tissue in the necrotic core undergoes necrosis due to ischemic irreversible injury. In the meantime, cardiomyocytes are lysed by proteolytic enzymes and release large amounts of cytokines to recruit neutrophils which participate in cardiac repair (7). Subsequently, sterile inflammatory response is initiated and the necrotic core is eventually replaced by granulation tissue, resulting in a collagen-rich scar formation (8). As the process of myocardial fibrosis varies between individuals, patients suffer different early deleterious changes in ventricular structure and function before scar formation (9). Notably, post-infarction angiogenesis offers novel therapeutic perspectives for saving the surviving ischaemic myocardium (10). Previous study indicated that cardiac repair after myocardial infarction includes a endothelial angiogenic response that initially begins in infarct border zone and spreads to the necrotic tissue (11). Angiogenesis is characterized by the sprouting of new vessels from the pre-existing myocardial capillary network (10). The repair of microvascular injury is largely dependent on the angiogenic rather than the arteriogenic response (12). In the initial phase, when exposed to stimulation by angiogenic signals, such as vascular growth factors, coronary endothelial cells are relaxed and detached from the vessel wall. Then, degradation of the basement membrane and extracellular matrix forms a temporary stroma (13, 14). The above process allows the endothelial cells to migrate and adhere to each other and to the extracellular matrix, a stage known as the sprouting of the endothelium. Subsequent hydrolytic remodeling of the extracellular matrix catalyzed by various enzymes ensures the stability of the pericyte-coated neovascularization and helps it to integrate into the coronary microcirculatory network (15).

Despite PCI surgery and secondary prevention medications for coronary artery disease, the patient's necrotic myocardial cells failed to regenerate. The formation of scar tissue limits the contractility of the ventricles, which may still irreversibly lead to heart failure. Therefore, salvaging the infarct border zone myocardium, which still has partial physiological function, is essential for the repair of myocardial injury. In recent years, angiogenesis in infarct border zone have attracted interest in the cardiovascular field. And researchers aim to explore new therapeutic directions for improving impaired cardiac function after myocardial infarction. However, bibliometric studies in this area are still lacking. The objective of this research was to explore hot spots, trends, and potential therapeutic directions in the field of angiogenesis after AMI over the past 20 years through bibliometric analysis. Overall, our study may help to provide novel insights for future clinical practice regarding the therapeutic application of AMI.

## Methods

### Search methodologies and data collection

The database selected for data retrieval was the Science Citation Index Expanded (SCI-EXPANDED) from the Web of Science Core Collection (WoSCC) (12). Data was acquired from the aforementioned database on January 2nd. In the WOS database, the following keywords and strategies are used: #1, “angiogenesis”; #2, “acute myocardial infarction”; #3, “#1” AND “#2”. Articles from 2000 to 2024 (January 2nd, 2024) were identified. English was the selected language, and the document type included both articles and reviews. SCI-EXPANDED database was used to identify articles, and the associated data was entered into Excel 2019. The qualifying papers were analyzed independently by two writers. We exported the full record of data from WoSCC, including the number of annual publications; outputs of countries/regions, journals, authors and total citations; impact factor (IF) in 2022, Journal Citation Reports (JCR) 2022 and Hirsch index (H-index). All data were downloaded in TXT format, and renamed with the “download∗.txt” convention to ensure that they were read correctly by the CiteSpace and VOSviewer software.

### Bibliometric analysis

**Figure 1:**
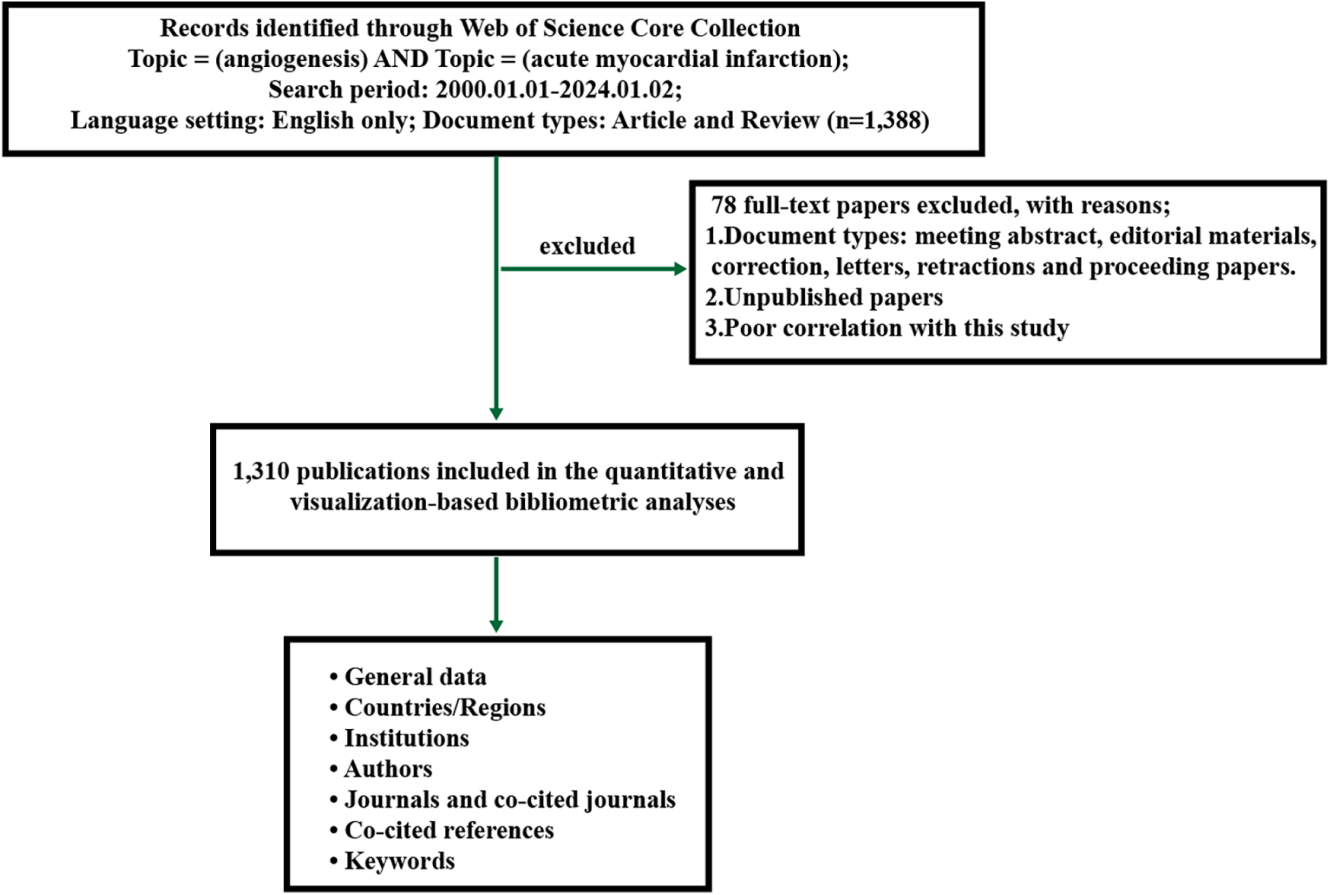
Flowchart for the research's search process.

Microsoft Excel 2019, VOSviewer, and CiteSpace were utilized for bibliometric and visual analysis of the relevant documents obtained from WoSCC. The standard competition ranking method determines the order of ranking. The ranking order is determined using the standard competition ranking method. Figure 1 illustrates the utilization of VOSViewer and CiteSpace for conducting bibliometric analysis and network visualization (12). Through the examination of important scholars' published keywords and key references, we have identified and determined the current research focal points and cutting-edge areas within this particular field.

The retrieved data were put into the CiteSpace 6.1. R2 software to produce visualized maps to analyse the cooccurrence, clustering, and bursts. The parameters of CiteSpace were set as follows: time slicing (from 2001-01-01 to 2024-01-02), years per slice (1), links (strength = cosine; scope = withinslices); pruning (pathfinder + pruning sliced network + pruning the merged network); and the term source and node types are flexible parameters according to specific needs. All the clusters were labelled by keywords, and the loglikelihood rate (LLR) was used as the clustering algorithm. Synonyms were combined in the obtained analysis map according to the actual situation.

Visualization of bibliometric network graphs was performed using VOSviewer v1.6.10.0 (13). To conduct this research, VOSviewer was employed to analyze organizations, authors and keywords. Publication numbers are represented by the size of nodes, the strength of relationships is indicated by the thickness of lines, and the era or cluster of publications is denoted by the color of nodes. We set the counting method as full counting; other thresholds were shown in the corresponding chapter. In the clustermap, the size of node reflects the co-occurrence frequencies, and the same color represents the same cluster; furthermore, the link indicates the co-occurrence relationship, and the thickness of the link depends on a calculated strength value, which is proportional to the number of publications two researchers co-authored or the number of publications in which two keywords occur together.

CiteSpace is furnished with cluster analysis and timeline perspectives, enabling the possibility of visually assessing knowledge domains (14). Keywords can be categorized and the most relevant hotspots for studies on angiogenesis and acute myocardial infarction can be discovered using cluster analysis.

## Results

### Distribution of countries/regions and institutions

**Figure 2:**
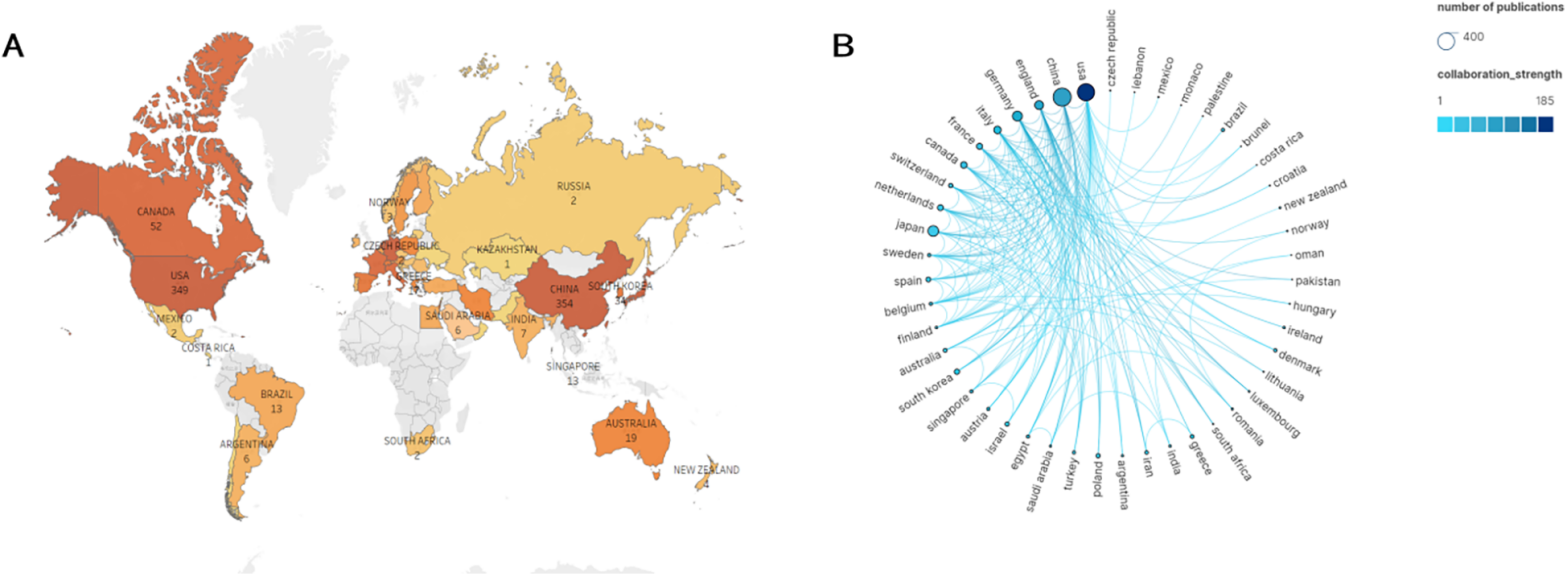
Geographical distribution of publications on angiogenesis in myocardial infarction research, 2000–2024. **(A)** Visualization of geographical distribution of the total number of papers on angiogenesis in myocardial infarction research from all nations and regions. **(B)** Analysis of national cooperation based on Scimago Graphica visual map. The nodes represent the number of publications. The larger the circle, the more publications in that country. The lines represent the frequency of academic exchanges between scholars from different countries. The darker the color of the line, the closer the cooperation.

Figure 2A shows that the 1,310 articles were published across 265 countries/regions. Several countries/regions collaborated on certain studies, relying on the statistical analysis (Figure 2B). According to the data presented in Table 1, the countries with the highest number of publications are China (354, 27.00%) and the United States (349, 26.62%). According to the analysis of centrality, from 2000 to 2024, the United States exhibited the greatest centrality score of 0.47, indicating that American scholars hold significant influence in this domain and maintain strong academic connections with researchers globally.

**Table 1:** Top 10 countries/regions and institutions related to angiogenesis in myocardial infarction research.

Rank	Countries/regions	Centrality	Count	Institution	Centrality	Count
1	China	0.11	354	Goethe Univ Frankfurt (Germany)	0.03	21
2	United States	0.47	349	Chinese Acad Med Sci (China)	0.09	21
3	Japan	0.01	135	Chang Gung Univ (China)	0.01	19
4	Germany	0.16	116	Nanjing Med Univ (China)	0.09	19
5	England	0.19	86	China Med Univ (China)	0.04	19
6	Italy	0.25	71	Chinese Acad Sci (China)	0.06	15
7	Canada	0.06	52	Peking Union Med Coll (China)	0.06	14
8	France	0.14	44	Capital Med Univ (China)	0.04	13
9	Netherlands	0.04	43	Univ Cincinnati (USA)	0.01	13
10	South Korea	0.08	36	Harvard Univ (USA)	0.05	13

**Figure 3:**
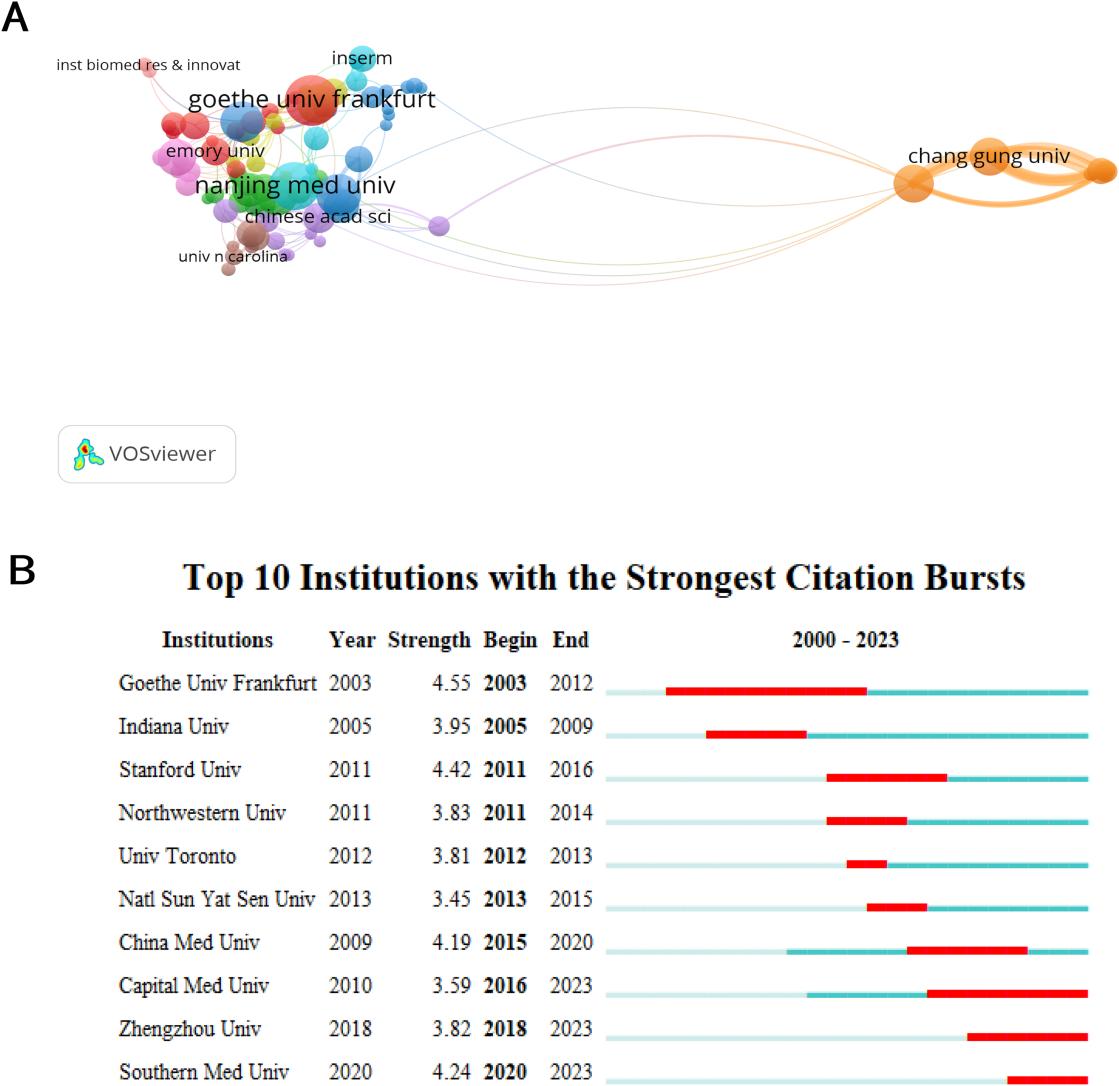
Coauthor analysis of organizations. **(A)** The institutions’ collaboration network visualization map generated by VOSviewer. **(B)** Top ten institutions with the strongest citation bursts by CiteSpace. ɣ: 1.0, minimum duration: 2.

Table 1 displays the top 10 most efficient organizations, comprising of seven Chinese institutions, two American institutions, and one German institution. Goethe University Frankfurt (21, 1.60%) and the Chinese Academy of Medical Sciences (21, 1.60%) stood out as highly productive institutions, highlighting their significant influence in the area of angiogenesis in acute myocardial infarction research (Figure 3A). The data indicates that Chinese Academy of Medical Sciences (0.09) and Nanjing Medical University (0.09) exhibit significant betweenness centrality, indicating their active engagement in collaborations with other institutions. Furthermore, it is evident that China's establishments exhibit a significant degree of collaboration, with fifty percent being ranked among the top 10 most productive institutions. By fully utilizing its regional advantages, China has acquired significant educational influence in this domain. Goethe University Frankfurt had the highest ranking for bursts among monitored institutions, with a burst period from 2003 to 2012 (Figure 3B). It was followed by Stanford University, which experienced a burst from 2011 to 2016, and Southern Medical University, which had a burst from 2020 to 2024.

### Analysis of authors

**Figure 4:**
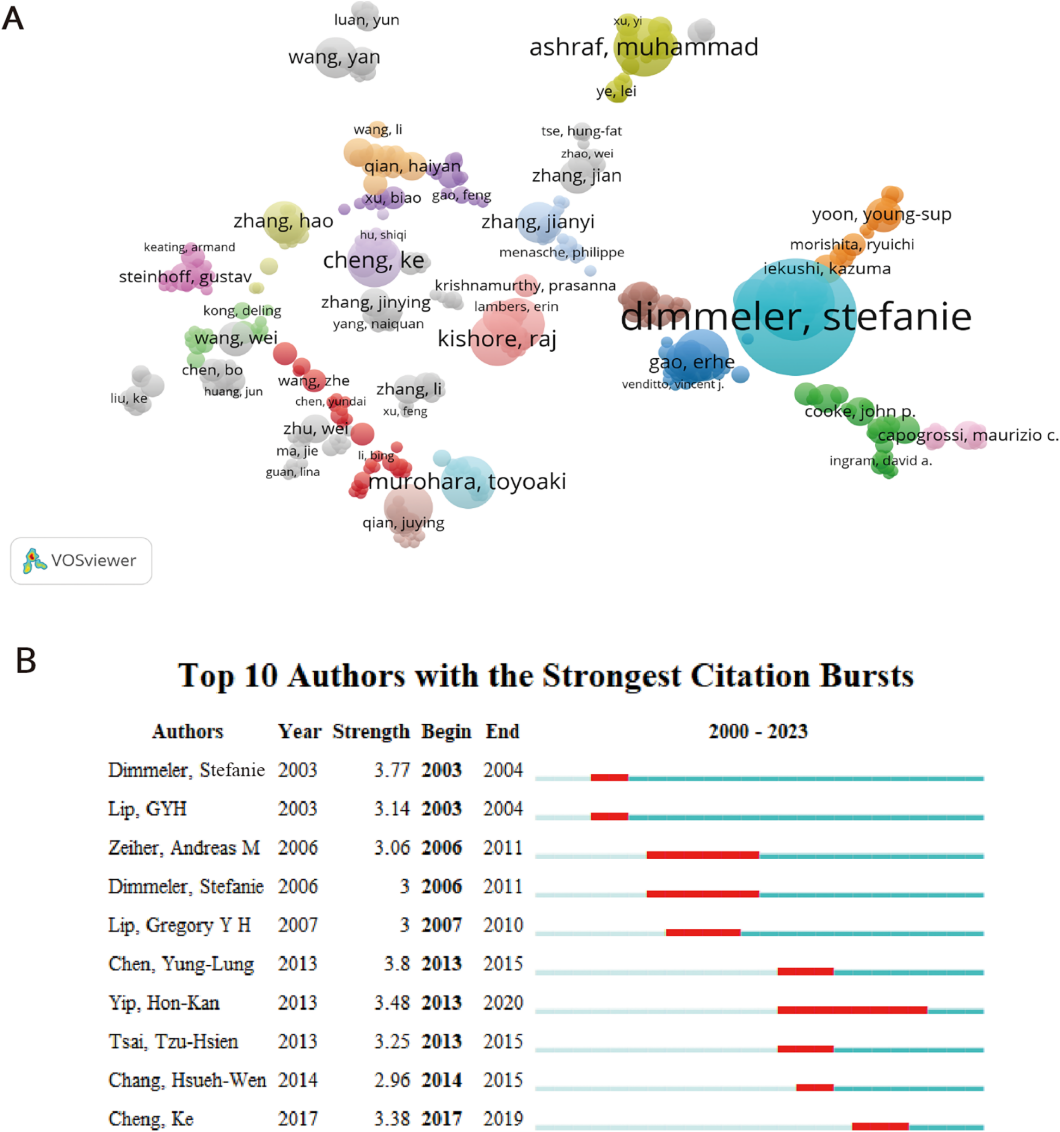
Coauthorship analysis of authors. **(A)** The analysis method was Linlog/modularity in VOSviewer, the weight was citations, and scores were the average published year. The color means the average published year. **(B)** Top 10 authors with the strongest citation bursts by CiteSpace. ɣ: 0.9, minimum duration: 2.

Analyzing core authors is beneficial for investigating the dispersion of documents. When evaluating primary authors, it is important to take into account factors like H-index and the overall count of citations. 8,099 authors published a total of 1,310 publications. From 2000 to 2023, Table 2 showcases a collection of the top ten primary contributors in this field. Stefanie Dimmeler holds the record for the highest number of published articles. Stefanie Dimmeler emerged as the frontrunner with 24 papers and a grand total of 66,942 citations. However, it was Gregory Yoke Hong Lip from the University of Livepool who claimed the top spot with an impressive total of 158,001 citations (*n* = 158,001) (Table 2). In Table 2, Gregory Yoke Hong Lip and Andreas M. Zeiher had h-index scores of 152 and 138, respectively. Figure 4A depicted the coauthorship relations of a thousand authors. Figure 4A displays the visualization map, illustrating the strong partnership among Stefanie Dimmeler, Ahmed Abdel-latif, and Young sup Yoon. Despite the gathering of many up-and-coming scientists (orange and yellow dots) and teams on the subject (Figure 4A), angiogenesis in AMI continues to be a significant field of fascination. Yung-Lung Chen achieved the highest ranking for author burst monitoring (Figure 4B), with Stefanie Dimmeler experiencing a burst between 2003 and 2004, and Hon-Kan Yip having a burst from 2013 to 2020.

**Table 2:** Core authors on angiogenesis in acute myocardial infarction research.

Authors	Affiliations	Documents	Total citations	Citations related to angiogenesis in AMI	h-index
Dimmeler, Stefanie	Goethe Univ Frankfurt	24	66,942	8,551	137
Andreas M. Zeiher	Goethe Univ Frankfurt	19	80,127	1,470	138
Yip, Hon-Kan	Chang Gung University	16	9,148	370	48
Sheu, Jiunn-Jye	Chang Gung Memorial Hospital	10	2,479	256	28
Sun, Cheuk-Kwan	I Shou University	10	6,097	288	42
Ashraf, Muhammad	Augusta University	10	7,601	517	46
Yang, Yuejin	Chinese Academy of Medical Sciences	10	6,017	363	37
Murohara, Toyoaki	Nagoya University	10	41,202	1,351	84
Chua, Sarah	Chang Gung Memorial Hospital	9	2,953	282	31
Lip, Gregory Yoke Hong	University of Liverpool	9	158,001	1,197	152

### Analysis of journals and and the top 10 high-cited articles

Among the 480 academic journals, the three most prominent scholarly publications are Circulation (occurring 46 times), Circulation research (occurring 37 times), and Plos one (occurring 28 times). Out of the top 15 journals (Table 3), the United States represented 46.67% (7/15), while England accounted for 13.33% (2/15). The top 15 active journals accounted for 24.79% of all publications, with a total of 325 articles being published. Among journals that published more than 14 articles, Circulation had the highest impact factor (IF 2022 = 39.918), closely followed by European heart journal (IF 2022 = 35.855) and Journal of the American college of cardiology (IF 2022 = 27.203). More than 520 citations were received by 50 journals, with Circulation (*n* = 1,199) and Circulation research (*n* = 1,015) being the journals with the highest number of citations. According to the findings in Table 3, a more detailed examination of the top 15 frequently referenced journals revealed that together they accounted for 32.82% of all citations.

**Table 3:** Top 15 journals and co-cited journals related to angiogenesis in acute myocardial infarction.

Rank	Journal	Count	IF(2022)	JCR(2022)	Cited journal	Cited count	IF(2022)	JCR(2022)
1	Circulation	46	39.918	Q1	Circulation	1,199	39.918	Q1
2	Circulation Research	37	13.081	Q1	Circulation Research	1,015	13.081	Q1
3	PLoS One	28	3.752	Q3	Journal of the American College of Cardiology	822	27.203	Q1
4	Journal of Molecular and Cellular Cardiology	27	5.763	Q2	Proceedings of the National Academy of Sciences of the United States of America	707	9.58	Q1
5	International Journal of Cardiology	24	4.039	Q2	Journal of Clinical Investigation	700	19.456	Q1
6	Cardiovascular Research	20	3.35	Q3	Cardiovascular Research	697	3.35	Q3
7	Stem Cell Research Therapy	18	8.088	Q1	Nature Medicine	664	87.241	Q1
8	American Journal of Physiology Heart and Circulatory Physiology	17	5.125	Q1	Nature	621	69.504	Q1
9	Journal of the American College of Cardiology	16	27.203	Q1	New England Journal of Medicine	609	176.079	Q1
10	Arteriosclerosis Thrombosis and Vascular Biology	16	10.514	Q1	Journal of Molecular and Cellular Cardiology	584	5.763	Q2
11	Journal of Cellular and Molecular Medicine	16	5.295	Q2	Lancet	581	202.731	Q1
12	Cell Transplantation	15	4.139	Q3	Science	577	63.714	Q1
13	Circulation Journal	15	3.35	Q3	European Heart Journal	569	35.855	Q1
14	European Heart Journal	15	35.855	Q1	Arteriosclerosis Thrombosis and Vascular Biology	527	10.514	Q1
15	Stem Cells	14	5.845	Q2	American Journal of Physiology Heart and Circulatory Physiology	526	5.125	Q1

**Figure 5:**
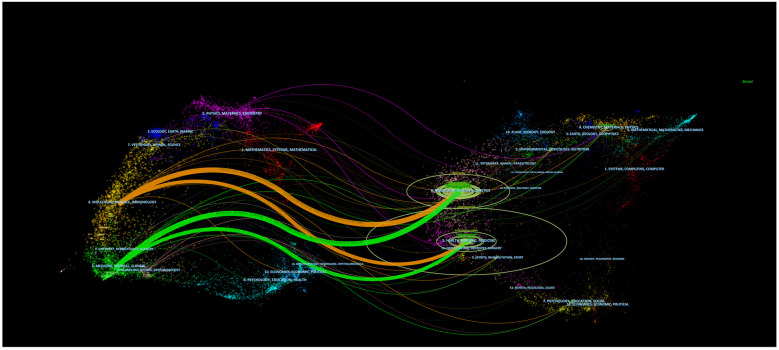
The dual-map overlay of journals on angiogenesis in acute myocardial infarction. The citing journals are on the left, the cited journals are on the right, and the colored path represents the citation relationship.

The overlay of journals in Figure 5, depicted in a dual-map format, illustrated the distribution of topics covered by the journals. All the journals cover different study areas, which are represented by the labels on the map. On the left side of the map, the journals that were cited appeared, whereas the journals that were citing appeared on the right side. Distinct colored lines represent the reference paths, starting from the citation map and ending there. The current map had four primary citation routes. Studies commonly referenced in the journals of Molecular/Biology/Immunology and Medicine/Medical/Clinical were published in the journals of Molecular/Biology/Genetics and Health/Nursing/Medicine. Table 4 comprises the ten most noteworthy studies that garnered significant interest from researchers. Circulation, in which three of the top ten extensively cited original publications were released, exert a notable scientific impact on academic achievement and experts in the domain. Notably, a 2005 study by Werner et al. (15) was given 1,726 citations. The article from Urbich et al. (16) received 1,491 times of citations.

**Table 4:** The top ten original articles with the most citations.

Title	First author	Journal	Year	Total Citations	Main conclusion
Circulating endothelial progenitor cells and cardiovascular outcomes	Werner, N	New England Journal of Medicine	2005	1,726	Endothelial progenitor cells derived from bone marrow are believed to support the integrity of the vascular endothelium. The number and function of endothelial progenitor cells correlate inversely with cardiovascular risk factors, but the prognostic value associated with circulating endothelial progenitor cells has not been defined. The level of circulating CD34 + KDR + endothelial progenitor cells predicts the occurrence of cardiovascular events and death from cardiovascular causes and may help to identify patients at increased cardiovascular risk (15)
Endothelial progenitor cells—Characterization and role in vascular biology	Urbich, C	Circulation Research	2004	1,491	Infusion of different hematopoietic stem cell populations and ex vivo expanded endothelial progenitor cells augments neovascularization of tissue after ischemia and contributes to reendothelialization after endothelial injury, thereby, providing a novel therapeutic option (16)
Autologous bone marrow-derived stem-cell transfer in patients with ST-segment elevation myocardial infarction: double-blind, randomised controlled trial	Janssens, S	Lancet	2006	996	The benefit of reperfusion therapies for ST-elevation acute myocardial infarction (STEMI) is limited by post-infarction left-ventricular (LV) dysfunction. They investigated the effect of autologous bone marrow-derived stem cell (BMSC) transfer in the infarct-related artery on LV function and structure. Intracoronary transfer of autologous bone marrow cells within 24 h of optimum reperfusion therapy does not augment recovery of global LV function after myocardial infarction, but could favourably affect infarct remodelling (17)
Mobilization of endothelial progenitor cells in patients with acute myocardial infarction	Shintani, S	Circulation	2001	929	Endothetial progenitor cells (EPCs) circulate in adult peripheral blood (PB) and contribute to neovascularization. This is the first clinical demonstration showing that lineage-committed EPCs and MNCCD34+, their putative precursors, are mobilized during an acute ischemic event in humans (18)
Physical training increases endothelial progenitor cells, inhibits neointima formation, and enhances angiogenesis	Laufs, U	Circulation	2004	678	Bone marrow—derived endothelial progenitor cells (EPCs) are thought to exert beneficial effects on atherosclerosis, angiogenesis, and vascular repair. Physical activity increases the production and circulating numbers of EPCs via a partially NO-dependent, antiapoptotic effect that could potentially underlie exercise-related beneficial effects on cardiovascular diseases (19)
Extracellular vesicles derived from human bone marrow mesenchymal stem cells promote angiogenesis in a rat myocardial infarction model	Bian, Suyan	Journal of Molecular Medicine	2014	471	Mesenchymal stem cells (MSCs) have been increasingly tested experimentally and clinically for cardiac repair. Their results suggested that like MSCs, MSC-EVs could also protect cardiac tissue from ischemic injury at least by means of promoting blood vessel formation, though further detailed investigations should be performed to define the functionality of MSC-Evs (20)
The immune system and cardiac repair	Frangogiannis, Nikolaos G.	Pharmacological Research	2008	471	Myocardial infarction is the most common cause of cardiac injury and results in acute loss of a large number of myocardial cells. Myofibroblast proliferation and angiogenesis result in formation of highly vascularized granulation tissue. Targeting inflammatory pathways following infarction may reduce cardiomyocyte injury and attenuate adverse remodeling. In addition, understanding the role of the immune system in cardiac repair is necessary in order to design optimal strategies for cardiac regeneration (21)
C-reactive protein attenuates endothelial progenitor cell survival, differentiation, and function—Further evidence of a mechanistic link between C-reactive protein and cardiovascular disease	Verma, S	Circulation	2004	448	Myocardial ischemia provides a potent stimulus to angiogenesis, and the mobilization and differentiation of endothelial progenitor cells (EPCs) has been shown to be important in this process. Human recombinant CRP, at concentrations known to predict adverse vascular outcomes, directly inhibits EPC differentiation, survival, and function, key components of angiogenesis and the response to chronic ischemia. This occurs in part via an effect of CRP to reduce EPC eNOS expression. The PPARgamma agonist rosiglitazone inhibits the negative effects of CRP on EPC biology. The ability of CRP to inhibit EPC differentiation and survival may represent an important mechanism that further links inflammation to cardiovascular disease (22)
Intravenous administration of mesenchymal stem cells improves cardiac function in rats with acute myocardial infarction through angiogenesis and myogenesis	Nagaya, N	American Journal of Physiology-Heart and Circulatory Physiology	2004	396	Mesenchymal stem cells (MSCs) are pluripotent cells that differentiate into a variety of cells, including cardiomyocytes and endothelial cells. Their results suggested that intravenous administration of MSCs improves cardiac function after acute myocardial infarction through enhancement of angiogenesis and myogenesis in the ischemic myocardium (23)
Atorvastatin enhances the therapeutic efficacy of mesenchymal stem cells-derived exosomes in acute myocardial infarction via up-regulating long non-coding RNA H19	Huang, PS	Cardiovascular Research	2020	365	Exosomes obtained from ATV-pretreated MSCs have significantly enhanced therapeutic efficacy for treatment of AMI possibly through promoting endothelial cell function. LncRNA H19 mediates, at least partially, the cardioprotective roles of MSCATV-Exo in promoting angiogenesis (24)

### Analysis of co-cited references

The top ten co-cited references related to angiogenesis in acute myocardial infarction are shown in Table 5. With 80 citations, Kai C Wollert had the most overall citations. Second place went to Volker Schächinger with 79 citations. The top ten journals with the highest impact factors were *Lancet* (IF 2022 = 202.731), *New England Journal of Medicine* (IF 2022 = 176.079), and *Nature Medicine* (IF 2022 = 87.241), according to the rankings.

**Table 5:** Top 10 co-cited references related to angiogenesis in acute myocardial infarction.

Title	First author	Journals	Citations	Type	Year
Intracoronary autologous bone-marrow cell transfer after myocardial infarction: the BOOST randomised controlled clinical trial	Kai C Wollert	Lancet	80	Clinical Trial	2004
Intracoronary bone marrow-derived progenitor cells in acute myocardial infarction	Volker Schächinger	New England Journal of Medicine	79	Randomized Controlled Trial	2006
Transplantation of Progenitor Cells and Regeneration Enhancement in Acute Myocardial Infarction (TOPCARE-AMI)	Birgit Assmus	Circulation	77	Clinical Trial	2002
Repair of infarcted myocardium by autologous intracoronary mononuclear bone marrow cell transplantation in humans	Bodo E Strauer	Circulation	66	Clinical Trial	2002
Intracoronary injection of mononuclear bone marrow cells in acute myocardial infarction	Ketil Lunde	New England Journal of Medicine	61	Randomized Controlled Trial	2006
Autologous bone marrow-derived stem-cell transfer in patients with ST-segment elevation myocardial infarction: double-blind, randomised controlled trial	Stefan Janssens	Lancet	52	Randomized Controlled Trial	2006
Transcoronary transplantation of progenitor cells after myocardial infarction	Birgit Assmus	New England Journal of Medicine	49	Randomized Controlled Trial	2006
Neovascularization of ischemic myocardium by human bone-marrow-derived angioblasts prevents cardiomyocyte apoptosis, reduces remodeling and improves cardiac function	A A Kocher	Nature Medicine	46	Article	2001
Angiogenesis in ischaemic myocardium by intramyocardial autologous bone marrow mononuclear cell implantation	Hung-Fat Tse	Lancet	45	Clinical Trial	2003
Haematopoietic stem cells do not transdifferentiate into cardiac myocytes in myocardial infarcts	Charles E Murry	Nature	45	Article	2004

**Figure 6:**
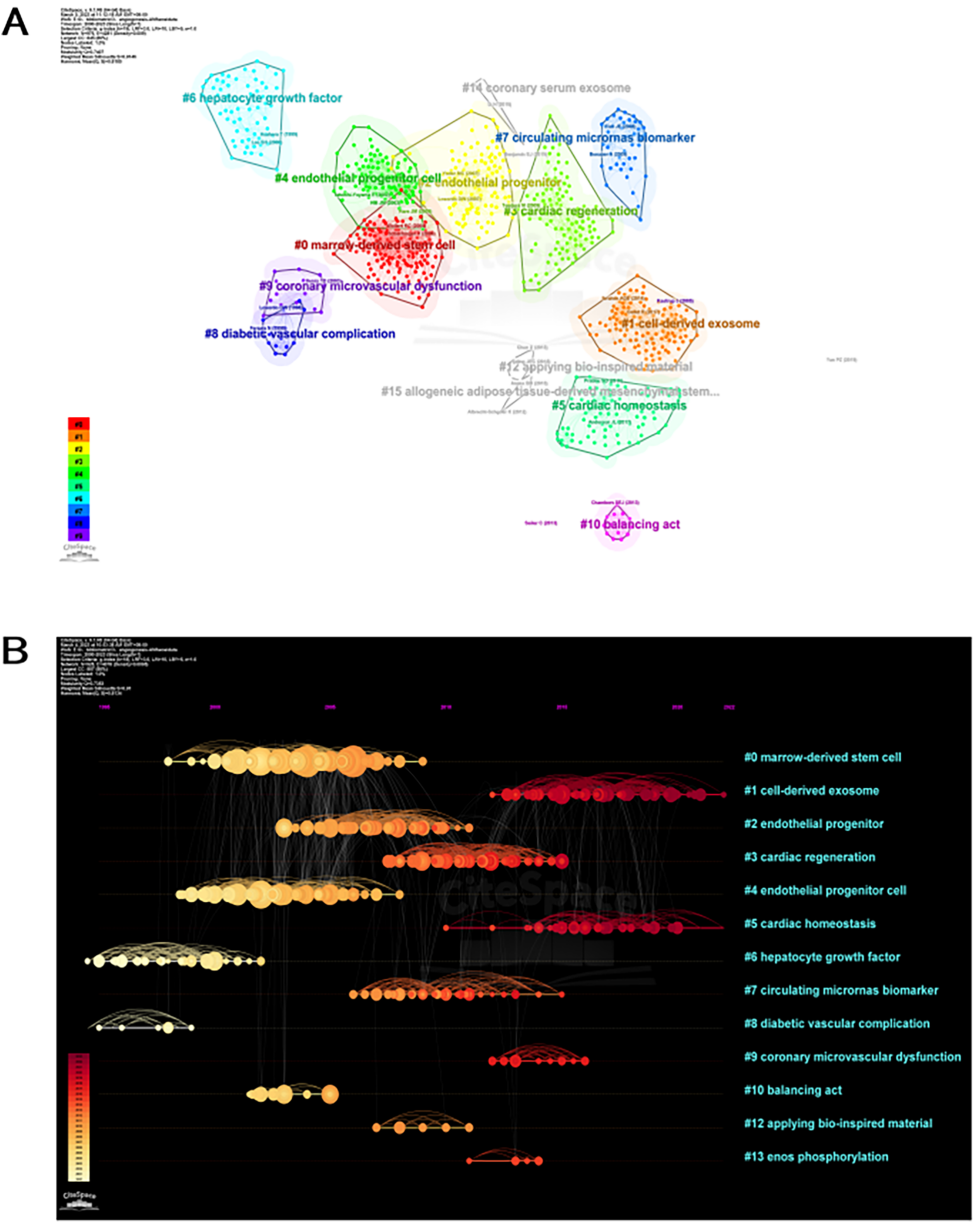
Citespace visualization map of cluster view. **(A)** Network map of co-cited references clusters. A cluster is assigned a tag number, and the smaller the number, the more closely related co-cited references comprise the cluster. **(B)** Timeline view of co-cited references related to angiogenesis in acute myocardial infarction. Each horizontal line represents a cluster; the smaller the number, the larger the cluster, and #0 is the largest cluster. The node size reflects the co-cited frequencies, and the links indicate the co-cited relationships; the color of node and line represent different years; nodes are at their first co-cited year. Cluster labels were extracted from title by LLR.

The study of the co-citation network is visually depicted in Figure 6A. The analysis revealed that the modularity Q achieved a value of 0.7407, while the weighted mean silhouette S showed a remarkable score of 0.9146, indicating a robust clustering effect and a uniform network. CiteSpace employed 45,911 co-cited references, employing a year as the temporal segment, encompassing the period from 2000 to 2023. The chosen subset consisted of the top 10% of extensively cited references, which were utilized to illustrate the co-citations (Figure 6B). Additionally, Figure 6B presents the co-citation references in a chronological view. The Figure 6B illustrates the evolution of research trends over time. According to the clustering results, it could potentially be divided into 13 clusters. A reference timeline was used to visualize areas of research hotspots. The cluster labels were assigned based on the most common terms found in each cluster. According to Figure 6B, cluster #0 (stem cell from bone marrow), #2 (progenitor of blood vessels), #4 (cell that gives rise to blood vessels), #6 (factor that promotes liver cell growth), #8 (complication of diabetes related to blood vessels), and #10 (process of maintaining balance) initiated earlier. On the other hand, cluster #1 (exosome released by cells) and #5 (maintenance of heart stability) are still in progress, indicating they are at the forefront.

### Keyword analysis

**Figure 7:**
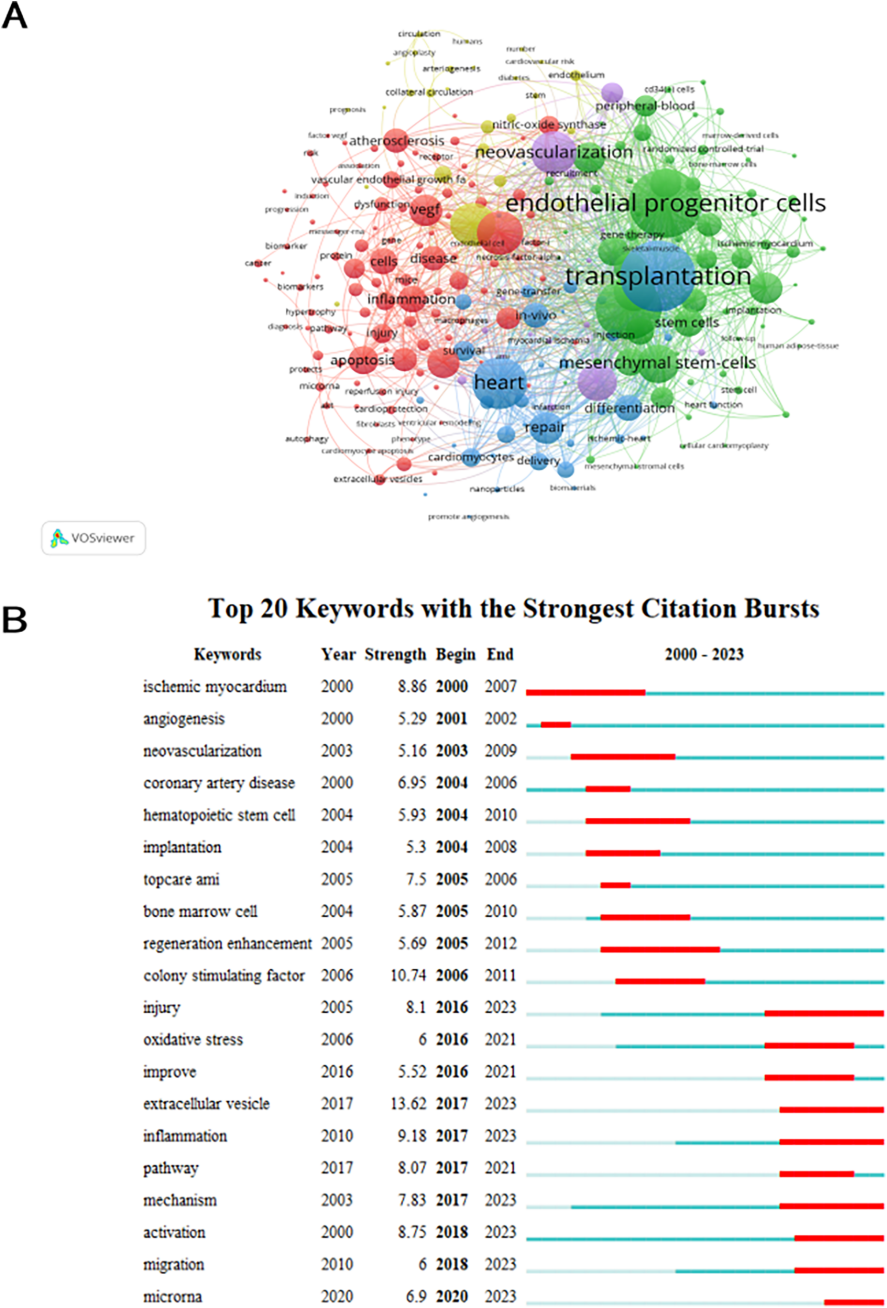
Analysis of keyword co-occurrence. **(A)** Keywords with documents ≥10 (cluster map). The size of node reflects the keywords’ co-occurrence frequencies, the link indicates the co-occurrence relationship between keywords, the thickness of the link is proportional to the number of publications two keywords have co-cited, and the same color of node represents the same cluster. **(B)** Top 20 keywords with the strongest citation bursts (sorted by the starting year). The red bars mean citation burstness.

A grand total of 4,384 keywords were extracted, with 261 occurring a minimum of ten times and 50 occurring a minimum of 50 times. With the exception of “angiogenesis” and “acute myocardial infarction”, it is evident that transplantation emerged as the predominant term (*n* = 228), closely followed by endothelial progenitor cells (*n* = 219) and progenitor cells (*n* = 197). Figure 7A displays overlay maps consisting of circles and labels to represent high-frequency keywords, with each cluster represented by a different color. Figure 7A displays five separate research directions represented by clusters that are colored red, green, blue, yellow, and purple. The main keywords of red cluster are endothelial growth factor, apoptosis, inflammation and atherosclerosis. The green cluster is characterized by the presence of endothelial progenitor cells, progenitor cells, and mesenchymal stem cells. Blue cluster is characterized by heart, cardiomyocytes and repair. Ischemia, blood flow, and arteriogenesis are the main focus of the yellow cluster. Purple cluster mainly include neovascularization, regeneration and mobilization. The intensity of the bursts in the keywords served as a notable indication of the frontiers, focal points, and developing patterns in the study as time progressed. Keyword bursts are frequently cited keywords that have been significantly mentioned over time. According to Figure 7B, the extracellular vesicle exhibited the most intense bursts (strength = 13.62), followed by colony stimulating factor (strength = 10.74) and inflammation (strength = 9.18). It is noteworthy that the study fields “injury” (2016–2023), “extracellular vesicle” (2017–2023), “inflammation” (2017–2023), “mechanism” (2017–2023), “activation” (2018–2023), “migration” (2018–2023) and “microrna” (2020–2023) are in burst until 2023, indicating that some study areas have recently been given considerable attention.

## Discussion

We analyzed and summarized the key contents covered by the original articles and review articles included in this study. Original research articles focus on emerging strategies for therapeutic angiogenesis after myocardial infarction, and scholars around the world are actively using cutting-edge molecular biology methods to explore different angiogenesis stimulation therapies to improve the long-term prognosis of patients with myocardial infarction. The important contents of original research articles are summarized as follows: Encouraging angiogenesis in the infarcted region is crucial for treating the aftereffects of AMI by reestablishing blood flow. In summary, these studies highlight various therapeutic strategies aimed at enhancing angiogenesis and improving cardiac function following acute myocardial infarction, including MSC therapy, exosomes, growth factors, and pharmacological agents. These approaches are underpinned by molecular mechanisms like VEGF signaling, miRNA regulation, and the SIRT1/mTOR pathway. Meanwhile, these studies investigate various therapeutic strategies to promote angiogenesis and improve heart function following AMI in animal models, focusing on different agents, such as traditional herbal remedies, Tie-2 receptors, low-intensity pulsed ultrasound (LIPUS) therapy, and bone marrow mesenchymal stem cell (BMEC)-derived exosomes (MSC-Exo). Gene therapy and biological factors are crucial in heart repair, with biological factors being more effective but having issues like a short half-life and easy inactivation. Overall, these studies demonstrate several promising approaches—growth factors therapy, gene therapy, biomaterials, herbal supplementation, Tie-2 modulation, MSC therapy, and exosome treatments—as viable options to enhance angiogenesis and mitigate heart tissue damage following AMI. There is a continued need to examine the therapeutic mechanisms driven by these novel approaches, especially when the molecules impacting the disease are not yet known. Even though there are several industrialization challenges for MSCs and exosomes, their potential in functional research, diagnosis, and treatment of diseases like AMI is evident. Additionally, there is still a great deal to discover about drug carriers and regenerative medicine and treatments, which have vast potential for growth in the future.

Review articles focus on the pathophysiological mechanisms of angiogenesis after myocardial infarction and its molecular signal transduction pathways. The key points of the review articles are summarized as follows: AMI causes significant damage to the coronary microcirculation, resulting in vascular rupture and capillary rarefaction. Tissue repair after AMI involves a strong angiogenic response that starts from the infarct edge zone and expands to the necrotic core area. Technological advances have provided new mechanistic understanding of angiogenesis after AMI, which may become a target for improving cardiac function. Studies have shown that new capillary structures are mainly formed by angiogenesis sprouting from existing endothelial cells (ECs) in the infarct edge zone, with little contribution from non-ECs. Single-cell RNA sequencing showed that ECs in infarcted hearts can be divided into clusters with different gene expression signatures, which may reflect functionally different cell populations. EC-specific multi-lineage tracing revealed clonal expansion of EC subpopulations after AMI. Tissue repair involves the interaction of multiple cell types, mainly through secreted proteins and their receptors. Although we are just beginning to understand the complexity of this intercellular communication, macrophages and fibroblast populations have emerged as the main drivers of the angiogenic response after AMI. Animal data support the concept of enhancing the endogenous angiogenic response after AMI to reduce scarring and adverse left ventricular remodeling. Thus, an improved understanding of infarct angiogenesis creates multiple therapeutic opportunities. In preclinical development, all proangiogenic strategies should be tested in animal models that replicate the cardiovascular risk factors and pharmacological treatments commonly prescribed to patients with acute MI. Given that most patients now do well after AMI, clinical translation will require careful selection of patients who require proangiogenic treatment.

### General information

The estimation of research advancements in angiogenesis in AMI can be based on the quantity and pattern of yearly publications. The study analyzed a total of 1,310 publications from 2000 to 2024, which consisted of 1,021 articles (77.88%) and 289 reviews (22.06%) sourced from 480 different journals. Based on CiteSpace and VOSviewer software, we implemented a bibliometric analysis to analyze studies related to the field of angiogenesis in AMI published in the WoSCC database. China has made the largest contribution to this field compared to many other countries. China, with a combined total of 354 articles, stands out as the most industrious nation due to its possession of four out of the five leading productive institutions (Chinese Academy of Medical Science, Chang Gung University, Nanjing Medical University, and China Medical University) and four out of the ten most productive researchers. Moreover, the close partnership between China and other nations has significantly contributed to the advancement of global communication and academic growth in this particular domain. Studying author collaborative networks helps comprehend influential scholars and the collaborative bond among researchers. Stefanie Dimmeler, hailing from Germany, emerged as the foremost author in terms of publication count. Stefanie Dimmeler's works have predominantly appeared in prestigious publications such as the *European Heart Journal*, *Circulation*, and *Nature Communications*. The most influential publication by Stefanie Dimmeler, titled “Endothelial progenitor cells: characterization and role in vascular biology”, was published in *Circulation Research* in 2004 (16). Their view showcased the crucial involvement of endothelial progenitor cells in neovascularization, also known as angiogenesis, among patients diagnosed with coronary artery disease. According to their report, it is possible that the mechanism is connected to the discharge of proangiogenic substances in a paracrine fashion, suggesting that the circulating cells that give rise to endothelial cells have the ability to save ischemic tissue (16). In 2006, Stefanie Dimmeler and his colleague, Gian Paolo Fadini, updated advances in the clinical application of endothelial progenitor cells in the treatment of ischemic heart disease in a review titled “Critical Reevaluation of Endothelial Progenitor Cell Phenotypes for Therapeutic and Diagnostic Use” in the *Circulation Research*. This study summarized the powerful differentiation capacity of endothelial progenitor cells allows them to display dynamic cellular phenotypes across time and space (25). In addition, a detailed biological functional characterization of endothelial progenitor cells using directed differentiation cell culture assays is meaningful, which can help to explore the potential of endothelial progenitor cells as biomarkers for predicting cardiovascular disease risk (25).

### Research hotspots and emerging frontiers

The term “Keywords with strongest citation bursts” refers to the timeframe in which the related research received extensive citations. It implies that the researchers have given careful consideration to the documents, potentially emphasizing the ongoing change and trends in this domain. Analysing the role of angiogenesis's involvement in AMI research can be achieved by identifying the strongest citation bursts associated with a specific keyword. In this case, CiteSpace primarily focused on the research topics of “injury”, “extracellular vesicle” and “inflammation”.

### Microvascular injury after AMI

Although PCI can open the lumen of narrowed or even occluded coronary arteries in time to restore myocardial perfusion and save ischemic myocardial tissue (3, 4). However, it has been shown that even after opening acute epicardial vessel occlusion, about half of the AMI patients suffered coronary microvascular dysfunction (CMD), which severely compromises the clinical benefit as well as the long-term prognosis of patients (26). CMD refer to the structural and functional impairment of coronary microvessels, resulting in reduced coronary blood flow levels and ultimately myocardial tissue ischemia in the donor region (27). According to prior research, PCI has been found to successfully achieve TIMI flow class III in 95% of STEMI patients. However, approximately 50% of patients are unable to restore proper perfusion in the distal coronary microcirculation, leading to the development of coronary microcirculation disorders. These disorders are associated with adverse prognostic events, including heart failure and cardiac death (28). The main manifestations of coronary microcirculatory ischemia/reperfusion injury are microvascular obstruction, cellular occlusion, and extravascular compression (e.g., edema and intramyocardial hemorrhage), which are mainly caused by atherosclerotic plaque occlusion in distal coronary arteries (29). Prolonged ischemia of the epicardial coronary arteries leads to irreversible damage to the coronary microvascular endothelium within the downstream perfused region (30). Animal studies conducted previously have indicated that the microvascular network in the boundaries of the infarct remains intact in terms of its fundamental physiological structure, while the microvascular network in the central region of the infarct has suffered complete destruction (31). It is striking that reactive oxygen species generated during ischemia-reperfusion and immune cell-derived pro-inflammatory cytokines in subsequent injury repair are both promoters of vascular endothelial cell injury and extracellular messengers that activate angiogenesis within the infarct margin (32, 33). Precise therapeutic approaches for endothelial cell damage in CMD is lacking, however, there is great anticipation for research on strategies to manage myocardial infarction with non-obstructive coronary arteries (MINOCA) and ischemia with non-obstructive coronary artery disease (INOCA) (34).

### Triggers of postinfarction angiogenesis

Hypoxia acts as a strong inducer of angiogenesis in development, the equilibrium of adult tissues, and various diseases. Hypoxia-inducible factors (HIF-1 and HIF-2) are strongly expressed in the endocardium and epicardium near the necrotic infarct center and infarct border zone, enhancing capillary density and blood flow in the infarct area to promote angiogenesis after infarction (35). Moreover, recruitment of a great amount of inflammatory cells is necessary for tissue lysis and resorption during the coagulative necrosis process of myocardial tissue in the infarct core (36). On the one hand, neutrophils, the earliest natural immune cells involved in the necrotic process, release large amounts of lysosomal enzymes to hydrolyze the organelles, subcellular organelles, and fragmented nuclei in the necrotic core and finally absorb them through the blood vessels and lymphatic vessels (36, 37). On the other hand, the infiltration of large numbers of leukocytes will also release large amounts of inflammatory factors that will further cause damage to the myocardial tissue in the infarct border zone, and even eventually cause ventricular remodeling and left ventricular dilatation (38). In addition, critical for angiogenesis, vascular endothelial growth factor alongside placental growth factor (PGF) play essential roles, with studies indicating that, following MI in rats, endothelial cells within the infarct area express VEGFR2 for at least 7 days (39).

### Applications of therapeutic angiogenesis in cardiac injury

The repair of post-ischemic injury also relies on angiogenesis, which involves the creation of new blood vessels from existing ones. As a cellular therapy to rescue ischemic myocardium, promoting angiogenesis is a potential way to improve left ventricular dysfunction after ischemic myocardial injury (40). This is achieved mainly through the stimulation of vascular endothelial cells within the infarct border zone to generate blood vessels. According to recent research, the primary physiological foundation of angiogenesis involves the stimulation, growth, and movement of vascular endothelial cells, along with the development and reinforcement of newly generated sprouts (12, 41). This is a complex multistep process involving multiple cells. When the myocardium is chronically ischemic, firstly, the small arteries in the coronary vascular network are anastomosed with each other (42). Then a new collateral circulation is established, which bypasses the myocardial tissue at the site of blood flow interruption. Eventually, the blood supply status of the distal ischemic tissue is improved (42). However, after myocardial infarction, the myocardium itself can trigger an angiogenic response that differs from the endothelial progenitor cell differentiation during embryonic development (43). Ischemic myocardium recruits endothelial progenitor cells to infiltrate the ischaemic zones, which subsequently differentiate into mature endothelial cells. In another regulatory mode, the myocardium regulates the adjacent endothelial cells by paracrine regulation, which proliferate into a new capillary network at the site of injury (44). However, the pro-angiogenic capacity possessed by ischaemic myocardium following myocardial infarction is limited (45). Therefore, the proangiogenic therapy of ischemic border zone is considered a promising therapeutic strategy to improve cardiovascular function after AMI. Analysis of key terms indicates that the research interest is gradually transitioning from treating cardiovascular conditions with mesenchymal stem cells (MSCs) to exploring the heart-protective functions and molecular mechanisms of MSC-exosomes.

### Properties of MSCs in biology and its role in ischemia

Adult mesenchymal stem cells (MSCs) are self-renewing and have multi-directional differentiation potential. MSCs, under appropriate induction conditions, have the ability to transform into different types of tissue cells including bone, cartilage, adipose tissue, tendons, muscles, and nerves (46, 47). Currently, stem cell transplantation is an important tool for therapeutic angiogenesis. Most recent cell transplatation technologies for treatment of ischemic diseases are still in preclinical research phase, including haematopoietic stem cells, vascular endothelial cells and MSCs (48). Notably, MSC has advantages such as easy to obtain, along with simple to amplify and easy to genetically modify, which makes them highly advantageous in the treatment of ischemic diseases (49). Based on their origin, MSCs are categorized into varieties such as those from bone marrow (BMMSCs), adipose tissue (ADSCs), and the umbilical cord (ucMSCs) (50). Previous genomic and proteomic analyses have shown that MSCs can secrete more than 200 pro-angiogenic substances, of which soluble vascular growth factors have received the most attention (51). The secretion of vascular endothelial growth factor (VEGF), basic fibroblast growth factor (bFGF), and hepatocyte growth factor (HGF) by mesenchymal stem cells (MSCs) is crucial for the migration of vascular endothelial cells and the formation of blood vessels (52). Gnecchi et al. illustrated that injection of MSC pretreatment medium into the residual surviving myocardial tissue of rats after myocardial infarction for 72 h reducted the infarct size of in rats and decreased the proportion of apoptotic myocytes (53). Kamihata et al. demonstrated that after MSC were transplanted into the ischemic myocardial region in AMI pigs for 3 weeks, capillary density in this ischemic region was increased and collateral perfusion was improved (54). Additionally, numerous recent studies have shown that MSCs primarily aid in restoring cardiac function through their paracrine effects, particularly involving the role of exosomes (55, 56).

### The role of exosomes in MI

Exosomes, varieties of extracellular vesicles, are formed by the release of intracellular endosomes into the extracellular compartment by cytosolic vomiting (57). They range from approximately 30–150 nm in diameter and have a cup-like morphology. Investigations have revealed that exosomes are packed with various bioactive compounds, including proteins, lipids, and genetic elements (58). They selectively attach their membrane proteins to ligands on the target cell membrane, facilitating intercellular communication through the transfer of bioactive molecules (59). At the same time, exosomes can help target-cells perform a large number of cellular functions, including cell proliferation or cell differentiation, angiogenesis, stress response and immune signalling (60). Chen et al. demonstrated that exosomes improved cardiac remodelling, inhibited collagen deposition, promoted angiogenesis and thus reduced infarction rate in infarcted rats (61). Geng et al. found that exosomes extracted from the serum of AMI patients were closely associated with the repair and regeneration of cardiac tissue after MI (62). Ribeiro-Rodrigues et al. illustrated that exosomes isolated by cardiomyocytes under ischaemic conditions exhibit high levels of MMP, which promotes endothelial cell proliferation and sprouting (63). In addition, their results showed that ischaemic-exosomes were relatively rich in miR-222 and miR-143, which exerted a partial pro-angiogenic effect after myocardial infarction (63).

### The mechanisms of MSC-exosomes in angiogenesis

Numerous studies have demonstrated that MSC- Exosomes are capable of suppressing programmed cell death (PCD) and fibrosis, promoting blood vessel formation, and ameliorating the ischemic myocardial microenvironment (64). Wang et al. found that MSC-EVs contain high levels of miR-210, and following treatment with MSC-EVs, there is a reduction in the expression of the target gene Efna3 in HUVECs. Therefore, the pro-angiogenic activity of the MSC-EVs might be linked to a mechanism dependent on miR-210-Efna3 (65). Another study discovered that transplanting exosomes containing miR-132 into the ischemic hearts of mice significantly promotes neovascularization around the infarcted area by targeting RASA1, thereby protecting cardiac function (66). Further research indicates that in HUVECs treated with ADMSC-Exosomes, there is an upregulation in the expression of the angiogenic genes Ang1 and FLK1, and a downregulation of the anti-angiogenic gene Vash1 and TSP1. Concurrently, miR-125a enhances angiogenesis through the suppression of the vascular growth inhibitor DLL4 (67). Experiments with a chronic ischemic pig model revealed that BMMsc-Exosomes enhance capillary and arteriolar growth by upregulating the MAPK and AKT/eNOS signaling pathways, which improves blood flow in ischemic myocardial tissues and increasing cardiac output (68). By targeting ADAMTS16, the upregulated miR-29b-3p delivered by BMMsc-Exosomes improves myocardial angiogenesis and remodeling in MI rats (69). Following myocardial infarction, miR-543 in exosomes derived from human mesenchymal stem cells targets COL4A1, thereby facilitating the proliferation, migration, invasion, and angiogenesis of heart microvascular endothelial cells (70). To conclude, there is no doubt that MSC-Exosomes have a profound ability to ameliorate cardiac function and advance angiogenesis.

## Limitations

This paper presents a bibliometric study on angiogenesis in AMI, which is being presented for the first time. However, it is important to acknowledge certain limitations. Initially, the deadline for the research investigation was established as January 2nd 2024; however, due to ongoing publications in the WOS database, new studies were excluded from the final bibliometric collection. Additionally, our research solely focused on articles and reviews due to limitations in methodology and software algorithms, thus excluding other forms of studies that explored angiogenesis in AMI. Furthermore, the variability in the quality of the publications contained within the WOS database, along with researchers' varying emphasis on different research focal points at different periods, could potentially diminish the precision of the topological analysis.

## Conclusion

A bibliometric analysis was conducted on the knowledge map, hotspots, and frontiers of AMI research related to angiogenesis. China and the United States remain leaders in this field, showcasing their scholars' remarkable academic impact and productivity. Stefanie Dimmeler from Goethe University Frankfurt was the main writer who exerted a significant scholarly impact on the discipline. The current research included important journals such as *Circulation*, *European Heart Journal*, *New England Journal of Medicine*, and *Journal of the American College of Cardiology*. Moreover, we determined the management strategies for microvascular injury after AMI and therapeutic angiogenesis as research spotlight in the future.

## Data Availability

The original contributions presented in the study are included in the article/Supplementary Material, further inquiries can be directed to the corresponding author.
